# External Control Viral-Like Particle Construction for Detection of Emergent Arboviruses by Real-Time Reverse-Transcription PCR

**DOI:** 10.1155/2019/2560401

**Published:** 2019-10-07

**Authors:** Ivo Alberto Borghetti, Miriam Ribas Zambenedetti, Luciana Requião, Deusilene Souza Vieira, Marco Aurélio Krieger, Rita de Cássia Pontello Rampazzo

**Affiliations:** ^1^Instituto de Biologia Molecular do Paraná (IBMP), Curitiba, PR, Brazil; ^2^Universidade Federal do Paraná, Programa de Pós-Graduação em Engenharia de Bioprocessos e Biotecnologia, Curitiba, PR, Brazil; ^3^FIOCRUZ, Rondônia, Porto Velho, Brazil; ^4^Instituto Carlos Chagas-ICC/FIOCRUZ, Curitiba, PR, Brazil

## Abstract

Arboviruses have been emerging and reemerging worldwide, predominantly in tropical and subtropical areas. As many arbovirus infections, including dengue (DENV), Zika (ZIKV), and chikungunya (CHIKV), have similar signs and symptoms, clinical diagnosis of arbovirus infections is challenging. Therefore, reliable laboratory tests are necessary to improve the clinical management of patients with suspected arbovirus infections. Real-time reverse-transcription PCR (RT-qPCR) is among the more effective methods to distinguish these viruses. The aim of this study was to construct a unique positive external control derived from a unique plasmid using genetic engineering for specific use in RT-qPCR assays to detect Zika, dengue (1–4), and chikungunya. An external control derived from the MS2 bacteriophage was constructed using sequences from arbovirus and human genomes. Laboratories were asked to test the control in the ZDC Biomol kit, a RT-qPCR kit which is able to detect Zika, dengue serotypes 1–4, chikungunya, and an internal human control. RNA extracted from the external control was able to be amplified and detected in RT-qPCR assays for each virus detected by using the ZDC Biomol kit. The external control, samples from viral culture, and infected patient samples display similar amplification using this assay. The pET47b(+)MS2-ZDC vector is a viable expression system for the production of external control viral-like particles (MS2-ZDC). The RNA from the recombinant particles can be easily extracted and can function as a tool to validate all steps of process from the extraction to the amplification of all targets in specific reaction. Thus, the MS2-ZDC particles are laboratory-safe in order to avoid risk for operators, and the phages are effective as positive control for use in the ZDC Biomol kit amplifying all kit targets making them effective for commercial profile.

## 1. Introduction

Emerging and reemerging viruses transmitted by arthropod vectors, such as yellow fever virus, Zika, dengue, chikungunya, Rift Valley fever virus, Japanese encephalitis virus, West Nile virus, Saint Louis encephalitis virus, Murray Valley encephalitis virus, Usutu virus, Spondweni virus, and O'nyong-nyong virus have transmission cycles in urban environments. These viruses cause overlapping clinical symptoms, and many patients develop serious physiological manifestations that can include death in severe cases [1, 2].

The anthropogenic environmental factors, such as disorganized urbanization, population displacement, and precariousness of basic sanitation, have favored the transmission and spread of these viruses. The last decade was chaotic in Brazil with a large number of infected people and many complications caused by these viruses, especially microcephaly (Zika), hemorrhagic syndromes (dengue), and severe arthralgias (chikungunya). This high prevalence brought loss for public health and country economy [3–5].

Since the clinical symptoms of arbovirus infections overlap, laboratory diagnostics are necessary to distinguish between them. Viral infections can be diagnosed through different methods including viral culture, serology, and molecular methods, and these same techniques are used to test for arbovirus infections [6, 7].

The advances in molecular methods are recognized in virology field. The qualitative molecular assays allow the early virus detection prior to the development of a detectable immune response or when it may be more difficult or impossible to grow in culture media or detect it by antigen tests. An early diagnosis can have a prompt significant impact on patient care [8].

Real-time reverse-transcription polymerase chain reaction (RT-qPCR) has been used to detect dengue (a virus that is part of family Flaviviridae, genus *Flavivirus*), Zika (a virus that is part of family Flaviviridae, genus *Flavivirus*), and chikungunya (a virus that is part of family Togaviridae, genus *Alphavirus*) worldwide in different samples such as serum, urine, cerebrospinal fluid, and saliva. RT-qPCR is easier to conduct than the other methods used to detect pathogens, and its quality depends specifically on the samples, the human operator, the nucleic acid test (NAT) kit, and PCR equipment [9–11].

So, our group developed a nucleic acid test called ZDC Biomol kit (Instituto de Biologia Molecular do Paraná, Brazil) for diagnosis of Zika, dengue serotypes 1, 2, 3, and 4, and chikungunya with an internal human control. This kit was used as a tool for Zika detection in saliva and urine from symptomatic patients. The results were validated with the extraction of RNA from viral cultures, which is a laborious method [7]. Furthermore, our product is incomplete, and in this article, we describe the development of a reliable positive control for this assay that is easy to produce and highly stable, presents low cost of production, and especially decreases the risk of cross contamination.

## 2. Materials and Methods

### 2.1. External Control Construction

The synthetic nucleotide sequence was designed based on targets of the ZDC Biomol kit (Instituto de Biologia Molecular do Paraná, Brazil). The synthetic sequence covers all specific targets that were analyzed using tools from the National Center for Biotechnology Information (NCBI). After the synthetic sequence was synthesized, it was cloned into the pET47b(+)-MS2 vector (GeneScript, USA). The derived vector (a unique plasmid with all targets) is referred to as pET47b(+)-MS2-ZDC.

### 2.2. External Control Production

The pET47b(+)-MS2-ZDC vector was transformed into NiCo21(DE3)-competent *Escherichia coli* in accordance with the manufacturer's instructions (New England Biolabs, USA). The protocol for expression and purification was described previously by Zambenedetti et al. [12], with some modifications. The expression of pET47b(+)-MS2-ZDC was induced by the addition of 0.5 mM isopropyl-1-*β*-D-thiogalactoside (IPTG), and after centrifugation, the supernatant was collected and processed with filtration for viral-like particles purification. After purification, viral-like particles were stored at –20°C.

### 2.3. Nucleic Acid Isolation

RNA was extracted from viral stocks obtained from infected cells (Zika, chikungunya, dengue 1, dengue 2, dengue 3, and dengue 4) and from MS2-ZDC controls with all targets for the 4 different reactions of ZDC Biomol kit. The extractions were done with aliquots of 140 *μ*L using the QIAamp viral RNA mini kit (QIAGEN®, Germany) in accordance with manufacturer's instructions. RNA was eluted at a final volume of 60 *μ*L.

### 2.4. Stability

The RNA was extracted from MS2-ZDC particles stored at –20°C for 0, 6, and 12 months.

### 2.5. Viability

MS2-ZDC particles were submitted to stress conditions before extraction. The particles were thawed and submitted to special temperature conditions: 37°C and 70°C twice for 1 or 2 hours. The RNA from these particles was then compared to RNA from particles which were not submitted to stress conditions.

### 2.6. Nucleic Acid Testing

Amplification was performed using 9.5 *μ*L of extracted ZDC-MS2 RNA and RNA from arbovirus cultures as a template in a 20 *μ*L final volume RT-qPCR to detect ZIKV, CHIKV, and DENV (serotypes 1–4) and an internal control using a 7500 Real-Time PCR Instrument (Applied Biosystems®) following the manufacturer's instructions ((51°C, 30 minutes) and (95°C, 15 minutes) followed 40 cycles (95°C, 15 seconds and 60°C, 60 seconds); the fluorescence was collected at 60°C).

The Biomol ZDC (IBMP) kit uses two duplex reactions and two triplex reactions in the same plate. One patient can be tested in four different assays: (1) Zika (FAM) and internal control (HEX); (2) Chikungunya (FAM) and internal control (HEX); (3) Dengue 1 (HEX), Dengue 4 (FAM), and internal control (Quasar 670); (4) Dengue 3 (HEX), Dengue 2 (FAM), and internal control (Quasar 670).

## 3. Results and Discussion

### 3.1. MS2-ZDC Production

We developed a positive external control based on a MS2 bacteriophage vector to be used in assays to detect the nucleic acids from Zika, chikungunya, and dengue virus serotypes 1–4. The pET47b(+)-MS2-ZDC vector was constructed after the insertion of a synthetic sequence into a unique BamHI site of pET47b(+)-MS2, which falls within the replicase gene of the MS2 genome. All of the constructs were confirmed by sequencing. pET47b(+)-MS2-ZDC could successfully generate MS2-ZDC particles whose genome harbored the synthetic sequence in the replicase gene. Consequently, a nonfunctional enzyme was produced, blocking replication of the genome.

### 3.2. MS2-ZDC Control in the ZDC Biomol Kit

**Figure 1:**
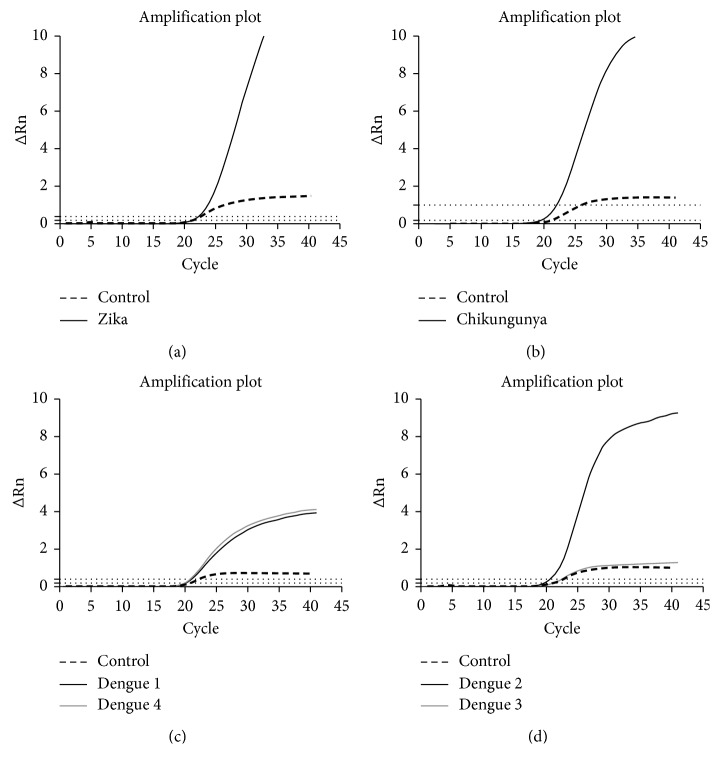
MS2-ZDC control RNA amplification plots in the ZDC Biomol Kit. MS2-ZDC control amplification in (a) duplex Zika/internal control reaction, (b) duplex chikungunya/internal control reaction, (c) triplex dengue 1/dengue 4/internal control reaction, and (d) triplex dengue 2/dengue 3/internal control reaction.

The RT-qPCR design for our experiments was composed of 4 different reactions which were able to detect (1) Zika, (2) dengue serotypes 1 and 4, (3) dengue serotypes 2 and 3, and (4) chikungunya, as well as a human transcript in each reaction as an internal control. RNA was extracted from MS2-ZDC particles and subsequently evaluated using the ZDC Biomol kit. The amplification results were equivalent to those obtained using samples from patients positive for each virus tested or RNA from virus culture (data not shown). So, this profile show that the particle construction, expression, purification, and RNA extraction were efficient presenting amplification for all targets in ZDC Biomol kit; our construction can be compared with a coinfected patient with all targets. The MS2-ZDC RNA control showed specific amplification for all targets in each reaction (four different reactions) of the ZDC Biomol kit and was expected in the same well in which the internal control was also amplified. The MS2-ZDC control amplifications are shown in Figure 1.

### 3.3. MS2-ZDC Particle Stability

To determine if the MS2-ZDC particles could be stored long-term and maintain stability, RNA extracted from MS2-ZDC particles stored at –20°C were assayed for Zika, chikungunya, and dengue 1–4 using the ZDC Biomol Kit. Aliquots stored for 6 or 12 months were compared with RNA from fresh particles which had not been stored (Table 1), and the results demonstrate no significant loss of Ct with prolonged storage. Additionally, the standard deviation was lower than 1 Ct. Therefore, the MS2-ZDC control particles can be stored at –20°C for up to 12 months without compromising their performance.

To further test the stability of the MS2-ZDC particles, we subjected them to temperature stress conditions as follows: 37°C and 70°C for 1 or 2 hours. After stress of the particles from which we extract RNA for assay by RT-qPCR, no significant difference in Ct values was seen when compared to particles stored –20°C. Therefore, the particles are quite stable, even under changing temperature conditions (data not shown).

## 4. Conclusions

Our study was focused on creating a positive control for a RT-qPCR-based diagnostic assay that detects Zika, dengue serotypes 1–4, and chikungunya for ZDC Biomol kit. We proposed an external positive control based on the MS2 bacteriophage modified. Generally, this strategy has been chosen when only one target is being assayed, but we developed our control to cover all seven targets in 4 multiplex RT-qPCR assays, based on the first control of our group that was used in HCV RT-qPCR assay [12]. The stability of our control can be highlighted because until now it was maintained for 12 months.

In conclusion, our study demonstrated successful construction, expression, and purification of a novel external control which can be integrated into the ZDC Biomol kit. Controls such as the one described here are especially important to ensure diagnostic RT-qPCR quality in the face of emerging outbreaks of Zika, dengue, and chikungunya. Furthermore, the development of the control was cost effective and less time consuming than the use of viral cultures.

We demonstrated the importance of the positive control in new discovery of our partners as presence of Zika virus RNA in urine and saliva from patients of Rio de Janeiro, Brazil, and the absence of RNA from Zika, dengue (serotypes 1–4) [9], and chikungunya virus from 676 serum samples of health blood donors from an endemic area in Bahia, Brazil [13], where the control was important for all steps from RNA extraction to amplification ensuring all processes and the first cases of Zika infection in Rondonia, Brazil.

The control was a fundamental point for development of a commercial research kit ZDC Biomol with all preparation steps performed in Instituto de Biologia Molecular do Paraná, Brazil. Currently, all laboratories operate under strict quality standards.

## Figures and Tables

**Table 1:** ZDC Biomol PCR results from MS2-ZDC particles under different storage conditions.

Reactions	Targets	0 months	6 months	12 months	SD
Duplex 1	Zika	22.19	23.16	21.47	0.85
	Control	21.38	21.96	21.99	0.35

Duplex 2	Chik	21.86	22.64	22.40	0.40
	Control	21.38	21.69	21.96	0.29

Triplex 1	Den1	20.10	20.98	20.91	0.49
	Den4	20.13	21.33	21.03	0.62
	Control	20.93	22.22	21.75	0.65

Triplex 2	Den2	20.54	21.20	20.94	0.33
	Den3	22.83	23.05	23.29	0.23
	Control	21.23	22.01	21.93	0.43

## Data Availability

All data used to support the findings of this study are available in the text and can be solicited from the corresponding author.
